# Work Experience and Anger Management in Nurses: Cross-Sectional Analysis Based on Benner’s Novice to Expert Theory

**DOI:** 10.2196/75432

**Published:** 2025-08-11

**Authors:** Donya Rahmati, Payam Nikjo, Hamzeh Zahabi, Zohreh Karimi, Leila Solouki

**Affiliations:** 1 Student Research Committee, Kermanshah University of Medical Sciences, Kermanshah, Iran; 2 Department of Nursing, School of Nursing and Midwifery, Kermanshah University of Medical Sciences, No. 72 Shahid Beheshti Blvd, Kermanshah, 6719851351, Iran, 98 9187167112, 98 8338211310

**Keywords:** anger management, work experience, Benner’s theory, emotional regulation, nurses

## Abstract

**Background:**

Nursing is an emotionally demanding profession where unmanaged anger can compromise patient care and teamwork. While clinical experience is thought to enhance emotional regulation, the relationship between work experience and anger management remains poorly understood.

**Objective:**

This study aimed to assess whether work experience predicts anger management ability among nurses, using Benner’s Novice to Expert Theory as a guiding framework.

**Methods:**

A descriptive cross-sectional study was conducted in 2024 involving 265 nurses working in hospitals affiliated with Kermanshah University of Medical Sciences, Kermanshah, Iran. Stratified random sampling was used based on hospital wards. Data were collected using a demographic questionnaire and the State-Trait Anger Expression Inventory-2. Statistical analyses included Pearson correlation analysis, *t* tests, ANOVA, and multiple linear regression analysis. Normality was tested using the Kolmogorov–Smirnov test. The sample size was determined using parameters referenced in prior studies and confirmed with G*Power software (Heinrich-Heine-University Düsseldorf).

**Results:**

Although nurses with more experience reported slightly higher anger control scores, the correlation between work experience and anger management was not significant (*r*=−0.079, *P*=.18). Regression analysis revealed that shift type and job security significantly predicted anger regulation, independent of experience level.

**Conclusions:**

Work experience alone does not ensure improved anger management among nurses. Organizational factors such as shift scheduling and employment stability may have a greater influence on emotional regulation. Institutions are encouraged to provide structured support and stress management training, especially for early-career nurses.

## Introduction

Anger is a fundamental human emotion that often arises in response to perceived threats, injustice, or high-stress environments. In the nursing profession, where emotional labor is constant and high-stakes decisions are routine, the ability to regulate anger is not just a personal asset—it is a professional necessity. Poor anger management in clinical settings can compromise patient safety, disrupt team dynamics, and ultimately lead to burnout and attrition [1].

Burnout, particularly its emotional exhaustion component, is widely prevalent among nurses. Systematic reviews report that 31% of nurses experience moderate to severe burnout, especially in high-pressure hospital environments [2]. The severity of burnout typically manifests across 3 dimensions: emotional exhaustion, depersonalization, and reduced personal accomplishment [3]. These dimensions are known to impair emotional self-regulation and increase the risk of reactive behaviors such as unregulated anger [4].

Despite the clinical importance of emotional regulation, including anger control, the factors that shape these abilities in nurses are not fully understood. While many assume that clinical experience enhances emotional resilience, research on the relationship between work experience and anger management has produced inconsistent findings [5]. Some studies indicate that experienced nurses exhibit better self-control and composure under pressure [6], while others suggest that accumulated stress may actually erode emotional regulation over time [7]. This inconsistency highlights a critical gap in the literature that merits focused investigation.

Benner’s Novice to Expert Theory offers a well-established framework for understanding professional development in nursing [8]. The model describes 5 progressive stages—novice, advanced beginner, competent, proficient, and expert—each characterized by increasing autonomy, situational awareness, and decision-making capabilities [9]. Importantly, Benner argues that these cognitive and clinical advancements are intertwined with psychological and emotional maturity [10]. As nurses advance in their careers, they are presumed to gain not only technical proficiency but also stronger coping mechanisms, including improved anger regulation [1].

This study is innovative in its integration of Benner’s theoretical stages with the construct of anger management—an intersection that has rarely been explored in empirical research. We aim to address this gap in the literature by investigating whether nurses’ anger control improves in tandem with their progression through Benner’s professional stages.

Based on Benner’s model, we hypothesize that nurses with greater work experience—corresponding to higher levels in the novice-to-expert continuum—will demonstrate significantly better anger management skills than their less experienced counterparts.

## Methods

### Study Design and Setting

This study used a descriptive cross-sectional design conducted in 2024 among nurses working in public hospitals affiliated with Kermanshah University of Medical Sciences, Kermanshah, Iran.

### Participants and Sampling

The study population consisted of clinical nurses working across multiple departments, including the emergency department, intensive care unit, surgical wards, and internal medicine units. A stratified random sampling technique was used, in which hospital wards served as the strata. Within each stratum, participants were randomly selected in proportion to the number of staff in each unit to ensure representativeness.

The inclusion criteria were being employed in a clinical nursing role at one of the participating hospitals, having a minimum of 6 months of work experience, being willing to participate and provide oral informed consent, and completing all study questionnaires.

Exclusion criteria were having a self-reported history of diagnosed psychological disorders, currently using mood-altering medications, and failure to complete the questionnaires.

These criteria were determined to increase internal validity and minimize confounding effects related to pre-existing emotional regulation deficits.

The minimum required sample size—computed based on a previous study assessing anger management in clinical staff [11], with an SD of 6.73 in anger control scores, and assuming a type I error of 5%, power of 80%, and effect size of 0.3—was determined to be 265 participants. G*Power software (version 3.1; Heinrich-Heine-University Düsseldorf) was used to verify this estimation.

### Data Collection Instruments

Two instruments were used for data collection: (1) a demographic questionnaire was used to collect data on age, gender, marital status, educational level, work experience (in years), shift type, hospital unit, and perceived job security; and (2) the State-Trait Anger Expression Inventory-2 (STAXI-2), developed by Spielberger, which consists of 57 items measuring multiple dimensions of anger including state anger, trait anger, and anger expression/control [12]. The Persian version of the STAXI-2 was validated by Asghari et al [13], with content validity confirmed by expert review and internal consistency measured by a Cronbach α of 0.84. In this study, the reliability of the instrument was reassessed and yielded an α coefficient of .85, confirming its suitability for the sample.

After obtaining ethical approval, the researchers coordinated with hospital administrators and distributed questionnaires in both printed and web-based formats. For the web-based version, participants accessed a secure form through the hospital’s intranet system using their unique staff codes to avoid duplicate entries. Data collection was conducted over a 3-week period.

### Data Analysis

Data were analyzed using SPSS (version 24; IBM Corp). Descriptive statistics (means, SDs, and frequencies) were reported for demographic variables. Inferential statistical analyzes included Pearson correlation (for relationships between continuous variables), independent samples *t* tests and 1-way ANOVA (to compare anger scores across demographic groups), and multiple linear regression (to identify predictors of anger management). Normality of the data was assessed using the Kolmogorov–Smirnov test. A *P* value of <.05 was considered significant in all analyses.

### Ethical Considerations

Prior to data collection, verbal informed consent was obtained from all participating nurses. The study was approved by the Research Ethics Committee of Kermanshah University of Medical Sciences (ethical approval code IR.KUMS.REC.1402.627). Participants were assured of the confidentiality and anonymity of their responses, and they had the right to withdraw from the study at any stage without any consequences. All participant data were fully anonymized. No compensation was provided to the participants.

## Results

### Anger Management Across Experience Levels

Participants were categorized into 5 groups based on Benner’s Novice to Expert theory. As shown in Table 1, there was a progressive increase in anger management scores across experience levels, from novice to expert. However, this trend was not significant.

A Pearson correlation test was used to examine the relationship between total years of work experience and anger management scores. The result showed a nonsignificant weak negative correlation (*r*=−0.079, *P*=.18).

**Table 1.** Mean anger management scores by experience level.

Benner level	Experience (years)	Nurses, n	Score, mean (SD)
Novice	0‐1	35	121.4 (15.8)
Advanced beginner	1‐3	82	129.2 (14.3)
Competent	3‐5	101	133.5 (12.7)
Proficient	5‐10	50	138.1 (10.9)
Expert	>10	22	142.7 (9.3)

### Comparative Analysis Based on Demographic Variables

An independent samples *t* test showed no significant difference in anger management scores between male (n=97) and female (n=168) participants (mean difference –2.5; *t*_261_=−1.32; *P*=.19). However, a 1-way ANOVA revealed a significant difference based on shift type (*F*_2,262_=6.12, *P*<.001), with nurses on rotational shifts having lower scores than those on fixed shifts.

### Multiple Regression Analysis

A multiple linear regression analysis was conducted to evaluate whether work experience, shift type, and job security predicted anger management. The model was significant (*F*_3,261_=8.47; *P*<.001; *R*²=0.22; Table 2).

Only shift type and job security were significant predictors. Work experience was not a significant predictor when other variables were controlled.

Although a descriptive trend indicated an improvement in anger management with more experience, the correlation was not significant. Rotational shifts and lack of job security were significantly associated with lower anger control. Work experience alone was not a reliable predictor when organizational variables were considered.

**Table 2.** Multiple linear regression predicting anger management.

Predictor variable	B (SE)	β	*t* test (*df*)	*P* value
Work experience (years)	−0.12 (0.09)	−.06	−1.34 (261)	.18
Shift type (rotational)	−5.27 (1.32)	−.21	−3.99 (261)	<.001
Job security (none)	−4.83 (1.11)	−.19	−4.35 (261)	<.001

## Discussion

### Principal Findings

This study aimed to explore whether work experience predicts anger management among nurses, framed within Benner’s Novice to Expert theory. While descriptive trends supported theoretical expectations—indicating higher anger control in more experienced nurses—the absence of statistical significance complicates a straightforward interpretation.

Benner’s model suggests that professional growth includes not only cognitive and technical advancement but also the evolution of emotional intelligence and situational awareness. From this perspective, an expert nurse should demonstrate greater emotional regulation than a novice [9,14]. However, our findings suggest that the translation of experience into emotional competence is not automatic, especially in environments marked by chronic stressors such as shift rotation and job insecurity.

The results echo those of studies showing that structural and organizational factors can override individual traits or experience. For instance, nurses working rotational shifts—regardless of their experience—reported significantly lower anger control. This aligns with research linking circadian disruption to poor emotional regulation [15]. Similarly, job insecurity was a strong negative predictor, supporting prior findings that emotional regulation is highly sensitive to perceived occupational stability.

Contrary to studies that have demonstrated a clear positive association between years of practice and anger control, our findings corroborate those suggesting that experience alone may be insufficient without parallel support systems [11]. This challenges the assumption that time in service naturally fosters better coping and invites a rethinking of how emotional skills are developed in clinical practice.

From a theoretical standpoint, our study highlights a gap between expected development (as described by Benner) and observed outcomes, which may be due to organizational neglect of emotional skill-building. If institutions fail to foster psychological safety, even expert-level nurses may struggle with emotional demands [16,17].

Our findings advocate for a dual-track model: one where clinical experience is complemented by intentional emotional training, such as structured anger management programs, mindfulness-based stress reduction, or resilience workshops. Moreover, policies aimed at reducing shift variability and reinforcing job security may prove more effective than relying solely on experience to shape emotional capacity.

### Limitations and Directions for Future Research

The cross-sectional design of this study limits causal inferences. Future longitudinal studies are essential to determine whether anger regulation skills improve over time or stagnate under persistent stressors. Additionally, emotional control was assessed using self-report instruments, which may not capture behavioral expressions or moment-to-moment reactivity. Future work should consider multimethod approaches, including peer assessment or biometric indicators.

Moreover, this study did not examine potential mediating variables such as emotional intelligence, personality traits, or organizational climate, which may shape the relationship between experience and emotional regulation. Exploring these variables through moderated regression models or qualitative interviews could provide richer insights.

### Conclusion

This study examined the link between work experience and anger management among nurses using Benner’s developmental framework. Although experienced nurses reported higher anger control scores, no significant correlation was found between years of practice and emotional regulation when organizational variables were controlled.

The findings suggest that professional maturity does not automatically translate into emotional regulation, especially in environments characterized by rotational shift work and job insecurity. These two factors were found to be stronger predictors of anger control than clinical experience itself.

This insight challenges traditional assumptions embedded in nursing education and workforce planning, which often presume that experience alone fosters emotional competence. Instead, the results advocate for a more intentional approach to emotional skill development within nursing practice.

Our key recommendation is that health care institutions should not rely solely on experience as a proxy for emotional regulation. Implementing targeted anger management training, promoting stable employment contracts, and reducing rotational shift burdens may yield greater improvements in nurse well-being and patient care outcomes [18].

## Abbreviations

STAXI-2: State-Trait Anger Expression Inventory-2
